# The Role of Epigenetic Modifications in Late Complications in Type 1 Diabetes

**DOI:** 10.3390/genes13040705

**Published:** 2022-04-15

**Authors:** Barbara Čugalj Kern, Katarina Trebušak Podkrajšek, Jernej Kovač, Robert Šket, Barbara Jenko Bizjan, Tine Tesovnik, Maruša Debeljak, Tadej Battelino, Nataša Bratina

**Affiliations:** 1 University Children’s Hospital, University Medical Centre Ljubljana, Zaloška Cesta 2, 1000 Ljubljana, Slovenia; katarina.trebusakpodkrajsek@mf.uni-lj.si (K.T.P.); jernej.kovac@kclj.si (J.K.); robert.sket@kclj.si (R.Š.); barbara.jenko.bizjan@kclj.si (B.J.B.); tine.tesovnik@kclj.si (T.T.); marusa.debeljak@kclj.si (M.D.); tadej.battelino@mf.uni-lj.si (T.B.); natasa.bratina@mf.uni-lj.si (N.B.); 2 Faculty of Medicine, University of Ljubljana, Vrazov Trg 2, 1000 Ljubljana, Slovenia

**Keywords:** type 1 diabetes, late complications, metabolic memory, epigenetics, DNA methylation, histone modification, microRNA

## Abstract

Type 1 diabetes is a chronic autoimmune disease in which the destruction of pancreatic β cells leads to hyperglycemia. The prevention of hyperglycemia is very important to avoid or at least postpone the development of micro- and macrovascular complications, also known as late complications. These include diabetic retinopathy, chronic renal failure, diabetic neuropathy, and cardiovascular diseases. The impact of long-term hyperglycemia has been shown to persist long after the normalization of blood glucose levels, a phenomenon known as metabolic memory. It is believed that epigenetic mechanisms such as DNA methylation, histone modifications, and microRNAs, play an important role in metabolic memory. The aim of this review is to address the impact of long-term hyperglycemia on epigenetic marks in late complications of type 1 diabetes.

## 1. Introduction

Type 1 diabetes (T1D) is one of the most common chronic diseases of childhood, however, it can be diagnosed at any age. T1D is an autoimmune disease characterized by the destruction of pancreatic β cells, leading to insulin deficiency [1,2]. The goal of the treatment is to reach average blood glucose levels below 8.6 mmol/L (154 mg/dL) and glycated hemoglobin (HbA1c) below 7% (53 mmol/L), and to spend more than 70% of the time in the glycemic range between 3.9 and 10 mmol/L (70 and 180 mg/dL) [3]. Keeping glycemic control within recommended targets is important in order to prevent or at least postpone the development of micro- and macrovascular complications, also known as late complications. These include diabetic retinopathy, chronic renal failure, diabetic neuropathy, cardiovascular diseases, and hearing impairment [2,4,5,6,7].

**Figure 1:**
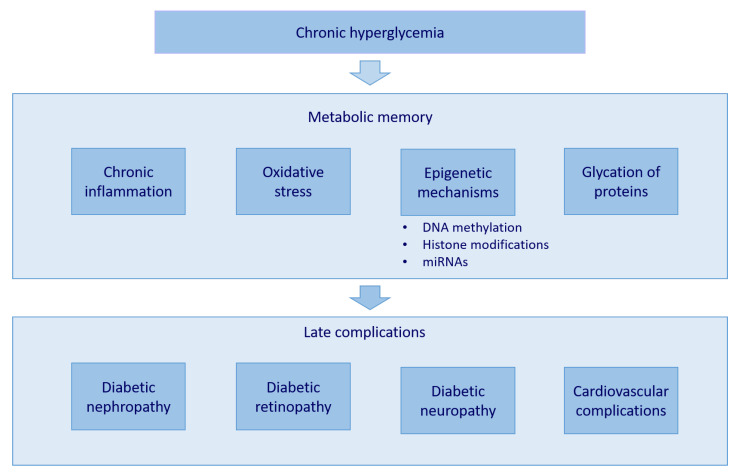
The metabolic memory occurs as a consequence of long-term hyperglycemia. Chronic inflammation, oxidative stress, glycation of proteins, and epigenetic mechanisms—namely, DNA methylation, histone modifications, and microRNAs—all contribute to its features. The consequence is the emergence of late complications, such as diabetic nephropathy, diabetic retinopathy, diabetic neuropathy, and cardiovascular complications.

Several clinical and animal studies demonstrated that the early establishment of intensive blood-glucose control reduces the risk for the development of late complications [8]. For example, a Diabetes Control and Complication Trial (DCCT) was carried out, in which conventional and intensive therapy were compared. The study showed that participants in conventional therapy had higher glycated hemoglobin (HbA1c) values than those in intensive therapy. At the end of the study, the participants in conventional therapy also adopted intensive therapy. HbA1c levels lowered and no difference was observed between groups [9]. In the follow-up study Epidemiology of Diabetes Interventions and Complications (EDIC), researchers discovered that participants in intensive therapy during the DCCT study had a lower incidence of late complications [4]. This phenomenon was called metabolic memory. It is believed that chronic inflammation, oxidative stress, glycation of proteins, and epigenetic mechanisms contribute to metabolic memory (Figure 1) [10].

**Figure 2:**
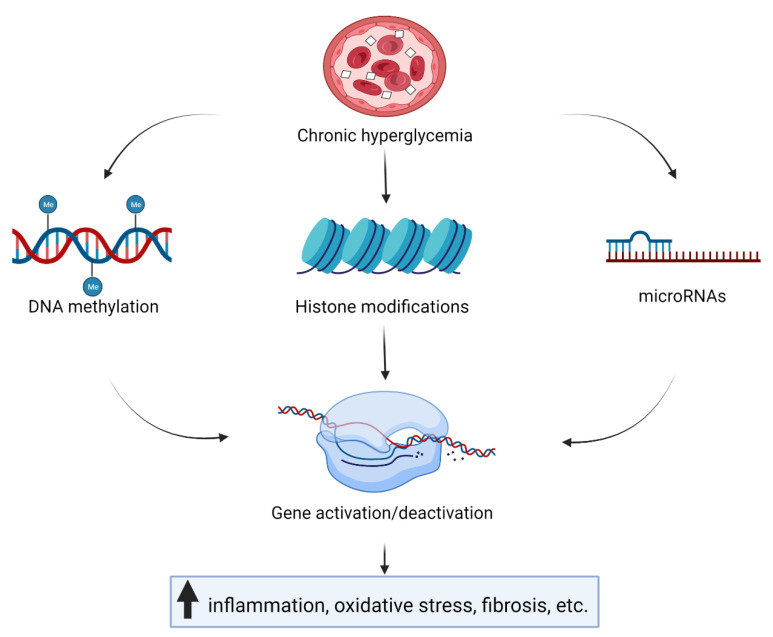
Chronic hyperglycemia influences gene transcription through epigenetic mechanisms—namely, DNA methylation, histone modifications, and microRNAs. The consequence is an increase in inflammation, oxidative stress, fibrosis, etc.

Epigenetic mechanisms—namely, DNA methylation, histone modifications, and microRNAs (miRNAs)—can influence transcription activity without changing the DNA sequence. They are essential for the regulation of all biological processes and are labile to environmental influences, such as nutrition, chemicals, stress, drugs, and infections [11,12]. Epigenetic changes have been associated with cancer [13], autoimmune and inflammatory diseases [14], cardiovascular diseases [15], obesity, and type 2 diabetes [16]. Furthermore, maternal nutrition during pregnancy was reported to influence fetal epigenetic modifications, which can cause a variety of long-term metabolic disorders in the offspring [17]. The aim of this review is to summarize discoveries of the impact of long-term hyperglycemia on epigenetic marks regarding late complications in T1D (Figure 2).

## 2. DNA Methylation and Late Complications

DNA methylation is a covalent modification of DNA involving the transfer of a methyl group to the fifth carbon of a cytosine residue to form 5-methylcytosine by DNA methyltransferases. Most DNA methylation occurs in a CpG dinucleotide context [18]. Demethylation is mediated through the family of ten-eleven translocase enzymes [19]. DNA methylation is essential for normal development, and it plays a very important role in several processes, such as the regulation of tissue-specific gene expression, genomic imprinting, X-chromosome inactivation, and silencing of repetitive element transcription and transposition [20]. Aberrant DNA methylation changes have been detected in several diseases [21].

DNA methylation analysis on blood DNA samples from DCCT participants showed that long-term hyperglycemia influenced DNA methylation. The association between the mean HbA1c value during DCCT and 186 CpG sites were identified. The persistence of DNA methylation patterns in monocytes collected 16 or 17 years later during the EDIC indicated a metabolic memory [22]. HbA1c levels and T1D duration were positively correlated with PhenoAge, a DNA methylation age calculator [23].

Different methylation patterns were observed between individuals with T1D with end-stage kidney disease and those without kidney disease in blood-derived DNA [24,25,26] and in DNA extracted from saliva [27]. Hypermethylation of genes was reported in blood-derived DNA in individuals with chronic kidney disease [26]. Global hypermethylation was also reported in peripheral blood mononuclear cells isolated from type 2 diabetes individuals with albuminuria [28]. Genome-wide DNA methylation analysis in peripheral-blood-cell-derived DNA from T1D individuals with and without diabetic nephropathy identified 19 CpG sites to be associated with the risk for the development of diabetic nephropathy. Among them, one CpG was located upstream of the transcription start site of the *UNC13B* gene, previously linked to diabetic nephropathy. Methylation levels on this CpG were higher in the case group [29]. Higher values of DNA methylation calculators, PhenoAge, and GrimAge were associated with a decrease in renal function [23]. Quantitative DNA methylation profiling in renal cell models showed that short-term exposure to high glucose levels is insufficient to cause alterations in DNA methylation profiles [30].

Genome-wide DNA methylation analysis in blood samples identified 349 CpG sites, corresponding to 233 genes, to be differentially methylated between individuals with proliferative diabetic retinopathy and individuals with long-term diabetes with no or mild retinopathy. Differences were observed in genes with known function in the retina and eye development, inflammation, diabetic complications, and oxidative stress. The majority of CpG sites showed decreased methylation in individuals with proliferative diabetic retinopathy. However, no difference in global methylation between the groups was observed [31]. In the retina of streptozotocin (STZ)-induced diabetic rats exposed to high glucose, hypermethylation was observed at the promoter region of POLG1, a catalytic subunit of the mitochondrial DNA replication enzyme. Hypermethylation persisted after the reversal of high glucose levels to normal levels. Similar findings were observed in retinal endothelial cells [32].

In peripheral blood cells of T1D individuals with cardiac autonomic neuropathy, increased DNA methylation was observed in the first intron of the *NINJ2* gene, coding a cell surface adhesion protein with a possible role in nerve regeneration. On the other hand, decreased methylation was reported in the first intron of the *BRSK2* gene, involved in neuronal polarization, and in the 5′ untranslated region of the *CLDN4* gene, coding a component of the tight junction [33]. A minor allele C of rs11085721 in the gene encoding *DNMT1*, a deoxyribonucleic acid methyltransferase, was associated with the risk for cardiac autonomic neuropathy but not for peripheral neuropathy in women with T1D [34]. The age calculator GrimAge was linked with the development of diabetic peripheral neuropathy and cardiovascular autonomic neuropathy [23].

Lower global DNA methylation was observed in diabetic foot ulcers fibroblasts and diabetic foot fibroblasts, compared with non-diabetic foot fibroblasts. DNA methylation patterns persisted even after multiple cell culture passages under normoglycemic conditions [35]. Furthermore, in animal models, global DNA hypomethylation induced by long-term hyperglycemia and its persistence in the normoglycemic environment were also identified in STZ-induced adult zebrafish [36] and STZ-induced rat bladder detrusor [37]. Genomic DNA hypomethylation was present in the liver but not in the kidney in diabetic rats [38]. In diabetic zebrafish, high glucose levels inducted the Parp family of enzymes, which led to stimulation of ten-eleven translocase enzymes, and the consequence was demethylation of high glucose-responsive loci, such as uhrf1, grtp1a, gcat, hnrnpa0, etc. [39].

## 3. Histone Modifications and Late Complications

Covalent posttranslational modifications of histones change histone–DNA interaction and thus influence gene transcription and other DNA processes, such as repair, replication, and recombination [40]. Different posttranslational modifications are known, such as histone acetylation, histone phosphorylation, histone methylation, and histone ubiquitination [41]. Histone acetylation and histone phosphorylation are normally associated with transcriptional activation. Histone acetylation occurs on the ε-amino group of lysine side chains. This causes the neutralization of the lysine’s positive charge, which weakens the interaction between histones and DNA. Histone phosphorylation also alters the charge of the histone protein. N-terminal histone tails of serine, threonine, and tyrosine are normally phosphorylated [42,43]. Histone methylation can lead to transcriptional activation or repression, depending on the site and level of methylation. Methylation occurs on the side chains of lysines, arginines, and histidines without affecting the histone charge. Lysines can be mono-, di-, or tri-methylated; arginines can be mono-, symmetrically, or asymmetrically di-methylated, whereas histidines can be monomethylated [44]. Histone monoubiquitination occurs on histone H2A at lysine 119 (H2AK119) and on histone H2B at lysine 120 (H2BK120). H2A monoubiquitination is linked to transcriptional repression, whereas H2B monoubiquitination is associated with transcriptional activation [45]. Aberrant histone modifications were linked to several different diseases and conditions [46,47,48].

In diabetic rat mesangial cells, the decrease in the H3 lysine 9 trimethylation 3 (H3K9me3) mark was found at promoter sites of pro-fibrotic genes such as *COL11A1*, *CTGF*, and *SERPINE1*, which contribute to the development of diabetic nephropathy [49]. The reduction in the repressive H3K27me3 mark was also identified at the promotor/enhancer sites of pro-fibrotic and inflammatory genes in diabetic rat renal mesangial cells [50]. Similar findings were observed in the kidneys of OVE6 mice and STZ-induced diabetic rats, together with increased levels of the activating epigenetic mark H3K4 demethylation [51]. In kidneys of STZ-induced diabetic rats treated with curcumin, increased acetylation of histone H3, together with increased dephosphorylation of histone H3, was reported [52]. High glucose levels influenced H2A and H2B monoubiquitination levels. In cultured glomerular mesangial cells, H2A ubiquitination was increased, and H2B ubiquitination was reduced [53]. However, H2AK119 and H2BK120 ubiquitination were increased in the whole kidney of diabetic animals, whereas in glomeruli of diabetic animals, their levels were reduced [54].

In vascular smooth muscle cells from diabetic db/db mice, decreased levels of H3K9me3 were identified at promoter sites of inflammatory genes—namely, *IL-6*, *MCSF*, and *MCP-1*. These aberrant chromatin changes persisted even after cells were cultured in vitro, indicating metabolic memory [55]. The persistence of the activating H3K4me1 mark was observed at the promotor site of the NF-κB subunit *p65* in aortic endothelial cells even when the cells were removed from the hyperglycemic environment [56,57]. Genome-wide histone H3K9/K14 hyperacetylation analysis in primary human aortic endothelial cells exposed to high or low glucose showed different histone acetylation patterns. The hyperacetylation was observed in regions of genes annotated to diabetes, coronary artery disease, and other cardiovascular diseases as a consequence of hyperglycemia [58].

Chronically high glucose levels caused changes in histone H3K4 and K9 demethylation in human monocytes [59]. In DCCT/EDIC participants, HbA1c levels were associated with monocytes H3K9 acetylation (H3K9Ac). Monocytes isolated from participants with a history of higher HbA1c levels had more promotor regions enriched with H3K9Ac. These promoters corresponded to genes related to numerous diabetes and diabetes complication-related pathways, including the TNFR2 signaling and the NF-κB pathway [60]. Miao et al. observed similar findings [61,62]. An in vitro study on human monocytes exposed to high glucose levels showed suppressed pro-inflammatory gene expression after the treatment with curcumin by decreasing histone acetylation [63].

In the diabetic retina, contradictory findings were observed. Histone hyperacetylation was observed in the retinas of diabetic rats, together with increased expression of inflammatory proteins [64]. On the contrary, in the retina of STZ-induced rats exposed to high glucose levels, the global acetylation of histone H3 was decreased, which persisted even after the reversal of hyperglycemia [65].

High glucose levels caused aberrant expression of various histone-modifying enzymes. Increased levels of SET7/9, a methyltransferase, stimulated the expression of extracellular matrix genes in renal mesangial cells [49,66,67], inflammatory genes in monocytes [68], and cultured vascular smooth muscle cells [55]. In renal mesangial cells, downregulation of *EZH2*, an H3K27me3 methyltransferase, and upregulation of H3K27me3 demethylases *KDM6B* and *KDM6A* were identified [50]. Levels of *SEV39H1* methyltransferase were decreased in diabetic conditions in vascular smooth muscle cells [55] and mesangial cells [69]. Single nucleotide polymorphism rs17353856 in the *SUV39H2* gene, a methyltransferase, was associated with diabetic retinopathy in individuals with T1D [70]. In cultured human monocytes, high glucose caused upregulation of *p300*, histone acetyltransferase and coactivator of NF-κB, and downregulation of histone deacetylase activity 2. These were reversed after the treatment with curcumin [63].

## 4. miRNAs and Late Complications

miRNAs are small ~22-nucleotide-long non-coding RNAs and negative regulators of targeted genes expression by binding to the 3′ untranslated region of mRNA [71]. They are involved in many biological processes, including cell differentiation, proliferation, cell metabolism, and apoptosis, and are often perturbed in many disease states [72]. Since they can be detected in plasma, urine, cerebrospinal, and other extracellular fluids, they can serve as potential noninvasive diagnostic biomarkers for many different diseases and conditions [73].

Increased levels of miR-21 [74,75,76,77], miR-135a [78], miR-192 [79,80], miR-200b/c [81], miR-214 [82,83], and miR-377 [84] were observed in diabetic kidney. All of them contributed to renal hypertrophy and fibrosis. miR-21 was shown to promote fibrosis of the kidney by targeting several metabolic pathways [74,75]. Dey et al. showed that miR-21 enhanced high glucose-induced TORC1 activity, leading to renal cell hypertrophy and fibronectin expression [76]. Inhibition of *SMAD7* and *PTEN* by miR-21 increased the expression of pro-fibrotic and extracellular matrix genes [77]. Decreased levels of *PTEN* were also associated with increased expression of miR-214 as a consequence of high glucose [82,83]. TGF-β-induction of miR-200b and miR-200c downregulated *FOG2*, an inhibitor of PI3K, thereby leading to activation of the PI3K–Akt pathway and glomerular mesangial hypertrophy [81]. Reduction of E-box repressors Zeb1/2 by miR-192 led to increased expression of collagen [79,80], and miR-200b/c [81]. miR-377 enhanced oxidative stress in mesangial cells and contributed to increased fibronectin protein production by targeting *SOD1*, *SOD2*, and *PAK1* [84].

On the contrary, levels of several miRNAs were decreased in the diabetic kidney, indicating their protective role in diabetic nephropathy [85,86,87,88,89,90]. The reduction in miR-25 resulted in increased levels of its target NOX4, resulting in increased generation of oxidative stress in mesangial cells [85,86]. Loss of miR-29b was linked to progressive diabetic kidney injury with microalbuminuria, renal fibrosis, and inflammations. Its direct targets are ColI/III/IV, fibrillin, and elastin [87]. The decrease in miR-146a correlated with increased albuminuria and glomerular damage, making it a potential biomarker for disease progression [88]. The expression of miR-424 in renal tissue of T1D rats was lower, compared with normal controls. Rictor was found to be its direct target [90].

In the retina of Akita mice, upregulation of miR-200b was observed. By downregulating its target *OXR1*, miR-200b was demonstrated to play a protective role against oxidative stress [91]. However, contradictory findings were identified in the retina of STZ-induced diabetic rats, as miR-200b was downregulated. The transfection of endothelial cells with miR-200b lowered the expression of VEGF mRNA and protein [92]. miR-451a was also downregulated in the diabetic retina. In diabetic conditions, miR-451a was identified to have a protective effect on mitochondrial function [93].

Levels of miR-39c were increased in the trigeminal ganglion tissue of STZ-induced diabetic mice. Targeting *Atg4B* miR-39c negatively affected the growth of trigeminal sensory neurons and the diabetic corneal nerve regeneration [94]. Upregulation of miR-341 and downregulation of let-7i were found in sensory neurons in mice with diabetic polyneuropathy. Restoring of let-7i or knocking down of miR-341 improved diabetic polyneuropathy phenotype [95].

Single nucleotide polymorphisms in miRNAs were associated with late complications. An rs2910164 polymorphism in seed sequence within miR-146a, leading to a reduction in mature miR-146a, was associated with diabetic nephropathy, and retinopathy [89,96]. In the southern Brazilian population, an association between the A allele of miR-126 rs4636297 and protection against diabetic retinopathy was observed [96]. miR-449b rs10061133 polymorphism was significantly associated with a decrease in risk for proliferative diabetic retinopathy, with a minor G allele being protective against diabetic retinopathy by altering a Dicer cleavage site [97].

A case–control study identified different plasma levels of miRNA associated with diabetic nephropathy. Levels of miR-25, miR-27a, miR-126, miR-130b, miR-132, miR-152, miR-320, miR-326, miR-340, and miR-660 were elevated, and levels of miR-181a, miR-223, and miR-574-3p were lowered. After pancreas–kidney transplantation, levels of miR-25, miR-27a, miR-130b, miR-132, miR-152, miR-320, miR-326, miR-340, miR-574-3p, and miR-660 were normalized [98]. In the plasma of individuals with severe diabetic kidney disease, miR-21-3p and miR-378-3p were upregulated, whereas miR-16-5p and miR-29a-3p were downregulated [99]. Circulating let-7b-5p and miR-21-5p were associated with the increased risk for the development of end-stage renal disease. Conversely, let-7c-5p and miR-29a-3p were associated with protection against rapid progression [100]. miR-21 could be used as a potential biomarker in peripheral blood for early detection of nephropathy in T1D since its levels start to increase within the first 5 years from the onset of diabetes [74]. In extracellular vesicles extracted from individuals with diabetic retinopathy, miR-150-5p was found to be downregulated, whereas miR-30b-5p was upregulated. miR-21-3p was also upregulated in samples of individuals with and without diabetic retinopathy [101]. Circulating miR-320a and miR-27b were linked to the incidence and progression of retinopathy [102]. miR-518-3p and miR-618 isolated from serum were positively associated with multiple microvascular complications: diabetic nephropathy, diabetic retinopathy, peripheral neuropathy, and cardiovascular autonomic neuropathy [103].

Urinary miRNA profiling in diabetic nephropathy linked 18 miRNAs with the development of microalbuminuria [104]. A set of 27 miRNAs was found in matched urine samples from T1D individuals at different levels in different stages of nephropathy [105]. Ghai et al. identified several changes in miRNAs concentration in urine and urinary extracellular vesicles between individuals with T1D with various grades of diabetic nephropathy or microalbuminuria [106]. In children and adolescents with T1D, urinary miR-377 levels were higher in individuals with microalbuminuria and positively correlated with HbA1c, carotid intimal thickness, and urinary albumin creatinine ration, while levels of miR-216a were lower and negatively correlated with these variables [107].

## 5. Future Perspectives

Establishing and maintaining normoglycemia after the onset of T1D is very important for preventing late T1D complications. The international study of glycemic control in people with T1D showed that glycemic control is still suboptimal [108]. The use of continuous glucose monitoring was shown to reduce glucose variability [109]. However, the discovery of biomarkers for the early detection of late complications is still crucial. A growing body of evidence suggests the correlation between long-term hyperglycemia and epigenetic modifications. Their persistence after the normalization of blood glucose levels indicates their involvement in metabolic memory, making them a vital area of research.

Epigenetic marks are good candidates as biomarkers for the early detection of late complications since they represent a link between the environment and gene expression [12] and are stable in both tissue and body fluids [110]. As opposed to genetics, epigenetic marks are mostly reversible; therefore, they could also be potential targets for novel therapeutic approaches [11]. However, due to the complexity of epigenetic marks, such as diversity in cell types [110], further studies are still necessary to elucidate the exact mechanisms of long-term hyperglycemia’s influence on epigenetic marks.

The use of the latest nucleotide sequencing detection techniques could give us new insights. Long-read sequencing enables the study of a wide range of modifications in single-molecule sequencing on native DNA. It can surpass traditional bisulfite sequencing for studying DNA methylation. Bisulfite sequencing, a golden standard for studying DNA methylation, is a DNA destructive method, and mapping generated short-read data to repetitive or low-complexity regions is very difficult [111]. The single-cell analyses allow the study of cell-specific epigenetic marks, compared with traditional profiling methods that analyze the bulk population of cells. Although these approaches are still under development, they will provide a more comprehensive understanding of how hyperglycemia influences epigenetic marks in different cell types [112,113,114]. Hopefully, knowledge of epigenetic modifications could contribute to the early detection of late complications and the possible development of targeted therapies.

## 6. Conclusions

A growing body of evidence shows the relationship between late complications of T1D and epigenetic mechanisms. Long-term hyperglycemia causes aberrant epigenetic marks that persisted even after the normoglycemic environment was established and maintained, indicating the involvement of epigenetics in metabolic memory. These marks can be used as biomarkers for the early detection of micro- and macrovascular complications. They may also serve as good targets for novel therapeutic approaches since epigenetic modifications can be reversed.

## Data Availability

Not applicable.
